# Sensorimotor dysfunctions as primary features of autism spectrum disorders

**DOI:** 10.1007/s11427-015-4894-4

**Published:** 2015-10

**Authors:** Matthew W. Mosconi, John A. Sweeney

**Affiliations:** Departments of Psychiatry and Pediatrics, University of Texas Southwestern Medical Center, Dallas, TX75390-9086, USA

**Keywords:** autism, motor, cerebellum, dyspraxia, oculomotor

## Abstract

Motor impairments in autism spectrum disorders (ASD) have received far less research attention than core social-communication and cognitive features. Yet, behavioral, neurophysiological, neuroimaging and histopathological studies have documented abnormal motor system development in the majority of individuals with ASD suggesting that these deficits may be primary to the disorder. There are several unique advantages to studying motor development in ASD. First, the neurophysiological substrates of motor skills have been well-characterized via animal and human lesion studies. Second, many of the single-gene disorders associated with ASD also are characterized by motor dysfunctions. Third, recent evidence suggests that the onset of motor dysfunctions may precede the emergence of social and communication deficits during the first year of life in ASD. Motor deficits documented in ASD indicate disruptions throughout the neuroaxis affecting cortex, striatum, the cerebellum and brainstem. Questions remain regarding the timing and development of motor system alterations in ASD, their association with defining clinical features, and their potential for parsing heterogeneity in ASD. Pursuing these questions through neurobiologically informed translational research holds great promise for identifying gene-brain pathways associated with ASD.

Motor disturbances represent a vastly understudied aspect of autism spectrum disorders (ASD). Yet, abnormal motor functions have been recognized in ASD patients since the original descriptions of these disorders by Leo Kanner [1] and Hans Asperger [2]. Recent systematic investigations indicate impairments in vestibular control, gross and fine motor movements, oculomotor functions and praxis in the majority of affected individuals (for a review, see [3]).

Despite these findings, motor dysfunctions are largely absent from current diagnostic criteria. DSM-IV TR includes motor stereotypies as one possible form of restricted and repetitive behavioral patterns that are core to ASD [4]. In the DSM, deficits of motor skills are described as features that may be associated with Autistic Disorder and Asperger’s Disorder. However, the descriptions do not specify the quality of motor impairments or integrate them into the diagnostic criteria. Rather it is indicated that “nonspecific neurological symptoms may be noted (e.g., primitive reflexes, delayed development of hand dominance) in Autistic Disorder” and “(in Asperger’s Disorder)…motor clumsiness and awkwardness may be present but usually are relatively mild”. In the context of data reported since the release of DSM-IV TR that has consistently identified motor disturbances in Autistic Disorder and Asperger’s Disorder, re-evaluation of the importance of motor skill deficits to ASD and their place in clinical diagnostic practice appears warranted.

In addition to their relevance to clinical diagnosis, motor functions offer several unique advantages for research studies examining the pathophysiology of ASD. First, motor systems lend themselves to translational research because their neurophysiological substrates are well-defined via animal and human lesion studies. Second, their spatial and temporal dimensions can be quantified precisely using readily available laboratory techniques, increasing the sensitivity of motor tasks to subtle deficits. Third, the limited language and cognitive demands of motor paradigms enable studies of individuals with a wide range of symptom severity and cognitive ability to be evaluated. Fourth, several studies now suggest that motor disturbances may be the earliest identifiable clinical abnormalities in ASD, and thus they could serve as markers of disease detectable in the first year of life [5–7]. Fifth, approximately 80% of genetic syndromes currently known to be associated with ASD are characterized by motor impairments [8]. Finally, a recent study indicated a unique pattern of oculomotor dysfunction in unaffected parents and siblings of individuals with ASD that were similar to abnormalities identified previously in affected individuals [9]. This suggests that certain oculomotor deficits and disruption of their underlying neural systems may be familial and specific to ASD.

## 1 Brain systems supporting sensorimotor functions

The human central nervous system consists of multiple interacting sensorimotor systems that involve cortico-cortical, cortical-subcortical, and cortico-cerebellar pathways that innervate lower motor neurons indirectly through the brainstem [10,11]. Relevant frontal lobe regions include primary motor cortex (Brodmann Area (BA) 4), lateral premotor cortex (BA 6) and the midline supplementary motor area (SMA; BA 6). These regions are directly connected with the intraparietal sulcus and inferior parietal lobule (BA 5 and 7), and this fronto-parietal system is involved in the planning and execution of accurate skeletomotor movements [12–14]. Frontal cortex-basal ganglia loops subserve the initiation and inhibition of voluntary motor acts [15,16], and disturbances to this system can cause failures to voluntarily and rapidly initiate movements, or to inhibit repetitive motor activity [17]. Cortico-ponto-cerebellar-thalamocortical loops involved in skeletomotor control primarily involve anterior cerebellar hemispheres (Lobules I–V and VIII) [18,19] that dynamically modulate motor performance to reduce deviations between intended and actual movements [20]. Lesions to anterior cerebellar lobules can result in uncoordinated and unsteady motor movements (for a review, see [21]).

**Figure 1:**
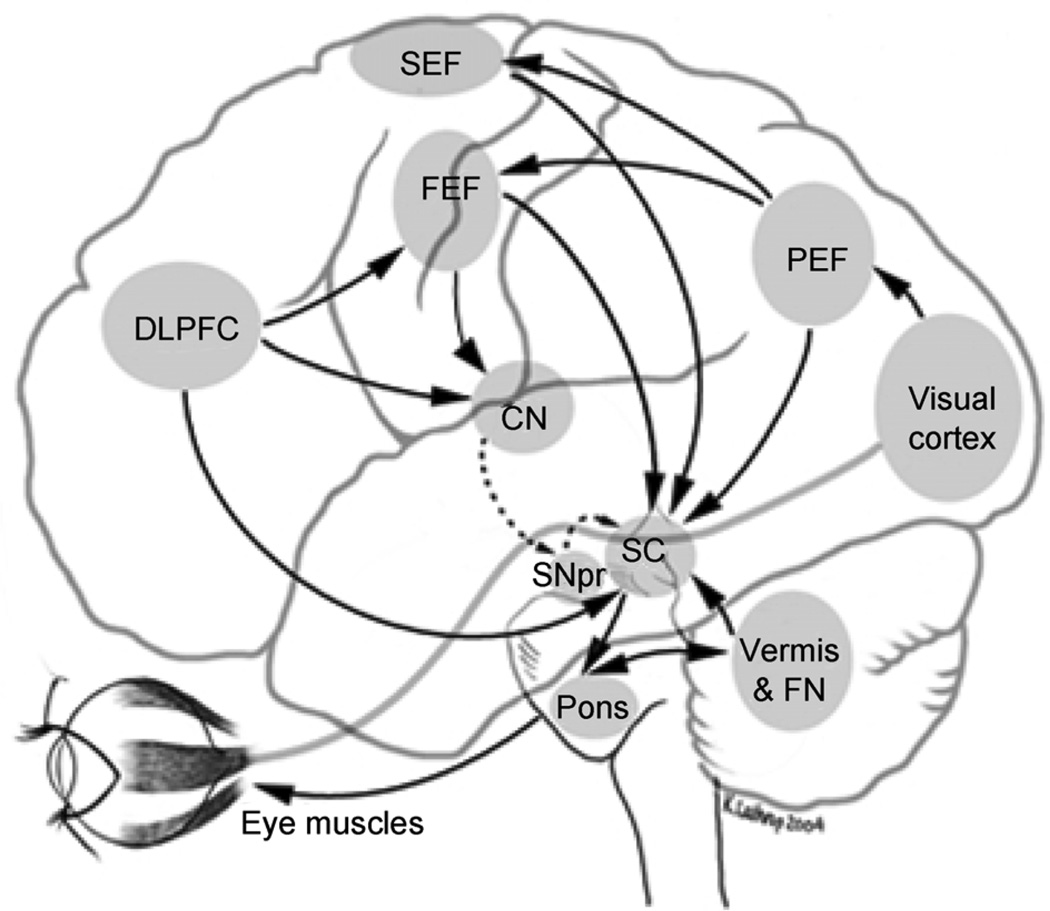
Lateral view of human cerebral cortex and projections to superior colliculus (SC) involved in saccade generation. Cortical and subcortical areas involved in oculomotor control, with excitatory and inhibitory pathways depicted in solid and broken lines, respectively. Direct excitatory pathways to the SC shown are from the dorsolateral prefrontal cortex (DLPFC), frontal eye fields (FEF), parietal eye fields (PEF), and supplementary eye fields (SEF). Indirect cortical input from the DLPFC and FEF is through the caudate nucleus (CN), which inhibits the substantia nigra pars reticulate (SNpr) and which, in turn, inhibits the SC. Cerebellar connections (vermis and fastigial nuclei (FN)) and pontine connections to the SC are also shown.

Like skeletomotor functions, eye movements rely on cortical and subcortical pathways. Visual information is processed in striate and extrastriate cortex (BA 17–19) and transformed into oculomotor commands via frontal and parietal cortices, the basal ganglia, brainstem and cerebellum (Figure 1). Oculomotor regions within the frontal lobe include the frontal eye fields (FEF) situated in premotor cortex (BA 6), supplementary eye fields (SEF) and preSMA positioned on the medial surface of the superior frontal gyrus (BA 6 and 8), and the middle frontal gyrus (BA 9 and 46) in dorsolateral prefrontal cortex (DLPFC). The FEF are involved in initiating rapid ballistic shifts in eye gaze (i.e., saccades) and slower velocity tracking movements (i.e., smooth pursuit eye movements) [22,23]. The SEF, in concert with DLPFC and parietal cortices, are involved in the preparatory phase of eye movements, sequencing multiple saccades, and coordinating eye and body movements [24]. The FEF and DLPFC are integrated in a frontostriatal system that includes anterior cingulate cortex (ACC) and the striatum. This system is involved in cognitive control of eye movements, including inhibiting context-inappropriate eye movements, error monitoring, short-term spatial memory and decision processes [13,25,26]. The parietal eye fields (PEF) are located in the intraparietal sulcus and parietal- occipital junction (BA 7 and 39) and are involved in generating conjugate eye movements and shifting visual attention [27,28]. Lobules VI–VII of the cerebellar vermis and output pathways via the fastigial nuclei act in concert with pontine nuclei and the superior colliculus to generate reflexive eye movements and calibrate eye movement accuracy [29].

Histopathological and neuroimaging studies of ASD each have documented abnormalities in motor structures. One of the first neuropathologic studies of an autistic brain described thickening of the arterioles and increased cell size in the right frontal lobe [30]. Neocortical pathology has since only been inconsistently documented (for positive findings, see [31], and for negative results, see [32]), but pathology within cerebellar circuits, first identified by Bauman and Kemper [32] has been consistently replicated [31,33,34]. MRI studies have identified cerebellar hypoplasia and, although this literature has been more equivocal than the post-mortem results [35–39], a meta-analysis of morphometry studies in ASD yielded significant overall reductions in cerebellar volume (Effect Size (ES)=0.72) [40]. This meta-analysis also documented cerebral (ES=0.62) and caudate nucleus (ES=0.4) enlargement in ASD. The most consistent MRI finding in ASD has been increased total brain volumes with diffuse increases in white and gray matter volumes across lobes and hemispheres (for a review, see [41]). Taken together, histopathological and morphometric studies suggest alterations that affect widely distributed brain systems in ASD involving cortical and subcortical structures integral to motor control.

## 2 Motor dysfunction in ASD and evidence of alterations in underlying brain systems

Fournier et al. [42] conducted a meta-analysis and reported large deficits in skeletomotor functioning in individuals with ASD (ES=1.2). The authors identified reduced postural stability, upper extremity dysfunction (e.g., poorly coordinated arm movements) and compromised movement preparation and planning. These findings suggest alterations to cortico-cerebellar, fronto-striatal, and fronto-parietal pathways. Additional studies of skeletomotor integrity in ASD have documented abnormal gait consistent with basal ganglia dysfunction [43,44] or cerebellar ataxia [45,46], imprecise and slowed fine motor movements [47–50], and dyspraxia [51,52]. Increased rates of mixed handedness also have been reported in ASD, suggesting reduced left hemisphere motor dominance [53–55].

Oculomotor studies of ASD indicate dysfunction throughout the neuroaxis. Reduced accuracy (dysmetria) of reflexive saccades [56] and increased trial-to-trial variability of saccade accuracy [57] suggest that the oculomotor vermis (lobules VI–VII), fastigial nuclei, and parapontine reticular formation are compromised. Reduced sustained smooth pursuit velocity [58] also is consistent with cerebellar dysfunction. Takarae and colleagues [58] reported lateralized alterations in the open-loop (first 100 ms) phase of smooth pursuit in which sensory feedback is not yet available to the visual system. While healthy controls showed greater rightward than leftward open-loop pursuit velocity, this asymmetry was not evident in patients. D’Cruz et al. [59] reported asymmetric procedural learning performance on a predictive saccade paradigm in which targets alternated at a fixed sequence between two predictable locations. While the one previous study using a similar procedural learning paradigm found bilateral impairments in ASD [60], the D’Cruz et al. findings, combined with findings of reduced rightward open-loop smooth pursuit gain [58] and reduced motor dominance in tests of handedness [53–55] indicate developmental dysmaturation affecting left hemisphere motor dominance.

Individuals with ASD also show impairments during tasks of cognitive control of sensorimotor responses. Cognitive control of eye movements has been assessed primarily with antisaccade and memory-guided saccade paradigms. Antisaccade paradigms require subjects to voluntarily inhibit a saccade towards a target and instead make a saccade away from the new target. Increased rates of antisaccade errors (eye movements towards rather than away from the target) have been consistently documented in ASD [56,60–63]. Mosconi et al. [64] found that antisaccade error rates were associated with higher-order repetitive behaviors in ASD, suggesting frontostriatal dysfunction may underlie behavioral rigidity. These brain systems develop throughout adolescence into early adulthood, and this protracted course of neural plasticity may provide a broad window for interventions aimed at increasing behavioral flexibility [56].

During memory-guided saccade tasks, subjects are required to make saccades to remembered target locations. Minshew et al. [62] demonstrated that adolescents and young adults with ASD had difficulty shifting their eyes accurately to remembered locations. Goldberg et al. [60] reported no abnormalities in the accuracy of memory-guided saccades in subjects with ASD, although they did report a prolonged latency of saccades to remembered targets. Luna et al. [56] also reported that latencies of memory guided saccades were longer in subjects with ASD than typically developing controls. Further, the ASD group’s age-related improvements were limited to childhood and early adolescence while no improvement was observed from adolescence to adulthood. In contrast, the control group showed progressive reductions in response latency through adulthood. This suggests an alteration in maturational processes that leads to a persistent impairment in the ability to quickly plan and initiate behavioral actions.

Functional neuroimaging studies of motor systems in ASD have identified several atypical patterns of brain function during motor control. Reduced activation within motor and lateral premotor cortex, SMA, and cerebellum has been reported during paced and sequential finger tapping tasks [65–67]. Studying both saccades and smooth pursuit eye movements, Takarae et al. [58,68] observed reduced activation within FEF, PEF and cerebellum. Interestingly, studies of manual and oculomotor functions each have documented increased activation in patients relative to controls within regions typically allocated to higher-level executive processing, including DLPFC, ACC, and the striatum [58,66,67]. While sensorimotor functions often involve automatic responses, they may occur at a more cognitive level in ASD and rely on higher order frontostriatal systems that are themselves impaired during cognitive tasks [56,60–63]. This compensatory use of frontostriatal systems to support sensorimotor functions may mitigate basic sensorimotor impairments, but consequently have adverse effects on later developing complex cognitive processes that rely on the ‘borrowed’ systems. Elucidating the developmental neurobiology of motor system deficits in ASD will require a mechanistic approach in which the neural system characteristics of component motor processes are investigated with longitudinal functional and anatomical neuroimaging and behavioral studies.

## 3 Is motor dysfunction a central feature of ASD?

The severity and pervasiveness of motor disturbances in ASD indicate that they may constitute a central feature. Yet, there remain significant gaps in our knowledge of motor dysfunction in ASD, including limited understanding of motor system development, the specificity of motor dysfunctions to ASD, and their interaction with other affected cognitive and behavioral systems. Developmental studies and studies comparing patients with non-ASD developmentally delayed and motor impaired subjects are needed.

Motor abnormalities in ASD appear to emerge within the first year of life [5–7,69,70]. Bryson et al. [5] reported that hallmark social-communication characteristics of ASD were largely absent at age 6 months despite motor impairment in seven out of nine infants studied. Motor impairments included poor visual tracking, limited motor control when grasping or reaching for objects and difficulty sitting independently. Additional motor disturbances were observed during follow-up at 12 and 18 months, including abnormal, repetitive motor behaviors and odd posturing. Interestingly, the two infants who did not show motor abnormalities by age 6 months did, in fact, demonstrate motor impairments by age 12 months. These infants were the only two infants studied who were later identified as ASD without meeting criteria on both the Autism Diagnostic Inventory–Revised [71] and Autism Diagnostic Observation Schedule [72]. Consistent with these data, retrospective analyses of home videotapes of infants later diagnosed with ASD have identified delays in motor milestones evident as early as 3–6 months [7,73,74]. Taken together, data from infants and toddlers later diagnosed with ASD have indicated that motor symptoms emerge early in ontogeny and may precede the development of core features.

There is little data on the maturation of motor systems in ASD. Fournier et al.’s meta-analysis [42] did not identify significant age effects on motor system deficits in ASD, although others have suggested that gross and fine motor abnormalities may diminish over the lifetime [47]. A cross-sectional study of oculomotor control by Luna and colleagues [56] has shown that sensorimotor impairments mediated by cerebellar-brainstem pathways may be stable during development or more profound during childhood (i.e., ages 8–12 years), whereas deficits in cognitive control of motor skills indicating frontostriatal dysfunction become more pronounced during adolescence or adulthood as healthy individuals develop skills that ASD patients do not. Both frontostriatal systems and the cerebellum develop throughout childhood and into adolescence, indicating that sensorimotor systems and their cognitive control may still be susceptible to interventions during childhood years [56,75–77]. Longitudinal behavioral and neuroimaging studies are needed to delineate patterns of motor and brain system dysmaturation in ASD, understand how these patterns differ from normative trajectories, determine if these impairments can be impacted by treatment, and identify patterns of developmental dysfunction across distinct motor processes and systems (e.g., eye movements versus limb movements).

Alterations of motor systems, like other clinical features of ASD, are of course not specific to ASD and therefore are not specific diagnostic indicators. Several studies have found that motor function in ASD cannot be distinguished from developmentally delayed non-ASD individuals. Provost, Lopez, and Heimerl [78] reported gross and fine motor delays in children with ASD ages 21–41 months but also indicated that these deficits were not disproportionate in children with ASD relative to children with specific motor delays. Baranek [74] observed abnormal postural behaviors in 9–12 month old children later diagnosed with ASD relative to typically developing children, but the children later diagnosed with ASD did not differ in their rate of abnormal posturing relative to IQ-matched infants. Thus, many of the motor problems in ASD may be sensitive but not specific markers for ASD-similar to other domains of deficit associated with the disorder when considered in isolation (e.g., repetitive motor mannerisms that also are present in non-ASD developmentally delayed individuals). Studies of dyspraxia and oculomotor dysfunction in ASD have studied only individuals without significant intellectual impairment; comparisons between cognitively impaired individuals with ASD and IQ-matched controls are needed to determine whether the magnitude or pattern of deficit distinguishes ASD.

Motor impairments that are specific to the disorder are of special interest, especially those that have not been reported for other developmental or neuropsychiatric disorders. In this regard, the observations of left-lateralized abnormalities on eye movement testing, for example reduced rightward open-loop pursuit gain [58] and alterations in rightward predictive saccades during a procedural learning paradigm [59], are particularly intriguing. Overreliance on proprioceptive relative to visual feedback during motor adaptation also may be specific to ASD [79]. Still, most studies of motor functions performed to date have not included cognitively impaired individuals with ASD, making it difficult to generalize findings regarding the integrity of motor systems to a broader population of affected individuals. While the requirements of functional imaging studies often preclude the inclusion of severely cognitively impaired individuals, novel strategies for acquiring anatomical data, including morphometric and diffusion weighted imaging, may provide a basis for useful studies of brain-behavior linkages across the autism spectrum and spectrum of cognitive ability.

Studies examining the relationships between motor impairments and other core features of ASD have indicated associations that may reflect common neuropathological substrates. Dziuk et al. [51] found that the level of dyspraxia in children with ASD was associated with their overall level of impairment in social, communication, and repetitive behavior domains. Similarly, Rogers et al. [80] reported that children with ASD who were “weak imitators” (defined as those performing below the mean level for age- and IQ-matched developmentally delayed children on tasks of imitation of sequential motor actions) had greater overall symptomatology, poorer social responsiveness, and weaknesses in fine motor skill relative to “strong imitators” with ASD. These studies suggest that either the core features of ASD result from a shared neurological basis with dyspraxia, or that impaired coordination of motor actions may contribute to the social and communicative deficits observed in ASD as well as patterns of repetitive behavior. Clearly dyspraxia is not a stand-alone cause of core features of ASD, as the majority of children with dyspraxia do not develop ASD. However, evidence that dyspraxia contributes to delayed development in oromotor and imitation skills integral to language and social development [81–83] and that the emergence of motor impairments often precedes that of social and communication impairments in ASD [5,6,74,84] together suggest that dyspraxia may be not only a central alteration in ASD but also a contributing factor to other impairments associated with the disorder [85]. Considerable development of the neurobiological systems associated with motor coordination and complex motor behavior precede the development of social and communication systems [86,87], consistent with the hypothesis that early motor system dysfunction may have downstream effects for developing social and communication systems in ASD. Non-motor brain systems have been shown to take on motor functions during childhood and adolescence in individuals with ASD [66,67,88] indicating that these regions may be developmentally compromised in part because they are called upon to provide ongoing compensation for deficits in motor skill. This is a novel and exciting hypothesis that may be important for understanding the sequential pattern of brain dysmaturation in individuals with ASD.

## 4 Do patterns of motor deficits distinguish ASD subtypes?

Asperger’s [2] original descriptions of his patients suggested that, in addition to exhibiting social impairments and a strong need for sameness, they showed a consistent motor “clumsiness”. Several studies have attempted to quantify this clumsiness and assess whether individuals with Asperger’s Disorder demonstrate reduced motor skill relative to both healthy individuals and individuals with Autistic Disorder. Findings from these studies have been inconsistent, demonstrating both more severe [89–91] and less severe motor deficits in Asperger’s Disorder [92,93]. Comparisons between these groups are difficult owing to the inconsistency of Asperger’s Disorder diagnostic criteria applied across studies [94]. Still, more recent electroencepholography (EEG) studies comparing individuals with Asperger’s Disorder to those with Autistic Disorder have indicated differential patterns of movement-related potentials (MRP). Rinehart and colleagues identified MRP abnormalities consistent with SMA-basal ganglia dysfunction during both externally [95] and internally cued [96] manual responses in individuals with Autistic Disorder, but not individuals with Asperger’s Disorder. Although these studies are based on relatively small samples (12–16 subjects per patient group) and the absence of an effect relative to controls in the Asperger’s groups could be a result of reduced power (as suggested by the authors), these findings do indicate that distinct pathophysiological profiles related to motor performance may distinguish ASD subpopulations. Such dissociations have been more equivocal in studies of social and complex neurocognitive functions suggesting that motor system studies hold promise for parsing heterogeneity in ASD.

Takarae et al. [57,68] compared individuals with ASD with and without a history of language delay (defined as no single words prior to age 2 years, or no phrase speech prior to age 3 years) on saccade and smooth pursuit eye movement paradigms and found that only individuals without language delays demonstrated saccade dysmetria [57], slower smooth pursuit latencies and failures to demonstrate the typical rightward directional advantage during smooth pursuit [68]. These results indicate distinct sensorimotor phenotypes that may be associated with different pathophysiologies manifest in early maturation of language skills. Research into possible mechanisms relating language development and sensorimotor skill is needed, but these studies are important in identifying potential sensorimotor signatures that could inform more reliable and biologically supported diagnostic subtypes.

## 5 Sensorimotor dysfunctions as intermediate phenotypes in ASD

An additional approach to parsing heterogeneity in neuropsychiatric disorders is to identify intermediate phenotypes that run in specific families and that can be linked to biological pathways of risk for illness. Such biological intermediate phenotypes should be found in affected and nonaffected family members at a higher rate than in the general population [97]. Studying 57 unaffected first-degree relatives of individuals with ASD, we recently identified a unique set of oculomotor impairments that are strikingly similar to those previously identified in individuals with ASD [9]. Deficits included saccade hypometria, increased trial-wise saccade variability, reduced closed-loop pursuit gain, reduced rightward open-loop pursuit gain, impaired response timing mechanisms for rightward predictive saccades, and increased rates of response suppression errors on an antisaccade task. These findings indicate that pontocerebellar circuitry alterations implicated by saccade inaccuracy, increased saccade amplitude variability and reduced pursuit gain may be familial. Similarly, rightward lateralized deficits in open-loop smooth pursuit and predictive saccade timing suggest left-hemisphere dysfunction affecting temporo-parietal and basal ganglia systems, respectively. Last, impaired antisaccade performance is consistent with frontostriatal abnormalities that also were implicated in the one previous study of eye movements in unaffected family members that identified inhibitory control deficits on a memory guided saccade task [98]. This profile of impairments, similar to those reported previously in independent samples, has direct implications for shared neurocircuitry dysfunction in individuals with ASD and their first-degree relatives. Future studies are needed to examine relationships between eye movement performance in probands and family members (these studies did not examine family trios), identify whether these impairments are heritable, and investigate gene variants in ASD that are associated with neurophysiological developments underlying saccade and smooth pursuit abnormalities. Still, this recent data indicating parallel eye movement alterations in independent sets of probands and family members suggest multiple distinct patterns of familial pathophysiology associated with sensorimotor disturbances.

## 6 Future directions

Research on motor systems in ASD is in its infancy and many questions remain unanswered. Are motor dysfunctions detectable during infancy and do they precede the emergence of core characteristics? How do these dysfunctions change over the course of development, and what can studies of this change tell us about altered brain maturation associated with ASD? Do studies of motor systems in ASD offer an advantage relative to studies of other core features for parsing heterogeneity via comparisons between diagnostic subtypes and assessment of unaffected family members? Studies investigating these questions hold great promise for earlier diagnosis, development of more individualized treatments, and generating pathophysiological and etiological models.

Integrating converging findings on the motor deficits in ASD into clinical diagnostic practice may be important for adequately characterizing the clinical phenotype(s), parsing heterogeneity and individualizing therapies. Recent evidence suggests that this area of deficit is central to ASD, although additional research into the specificity of motor and neurophysiological signatures in ASD is needed. In addition, parsing deficits in distinct motor systems may assist in localizing pathways of genetic risk. Few studies have systematically examined performance across distinct motor systems in the same subjects. Studying sensorimotor functions in unaffected family members has provided exciting results suggesting familial deficits affecting oculomotor systems. Neurophysiological studies of skeletomotor control in family members have not yet been performed but will be critical for localizing familial brain system disruptions. These studies could offer clues to pathophysiological mechanisms, indicate disrupted neural systems that co-segregate within families, and provide more homogeneous biological phenotypes for family genetic research.
